# Cancer patient perspective in the arena of COVID‐19 pandemic

**DOI:** 10.1002/pon.5774

**Published:** 2021-08-19

**Authors:** Zelmira Ballatore, Filippo Merloni, Nicoletta Ranallo, Lucia Bastianelli, Francesca Vitarelli, Luca Cantini, Giulia Ricci, Benedetta Ferretti, Paolo Alessandroni, Michela Del Prete, Silvia Chiorrini, Mobin Safi, Rita Ficarelli, Giovanni Benedetti, Luca Faloppi, Massimo Marcellini, Rosa Stoico, Rossana Berardi

**Affiliations:** ^1^ Clinical Oncology Università Politecnica delle Marche AOU Ospedali Riuniti di Ancona Ancona Italy; ^2^ U.O. Oncologia Ospedale B. Eustachio San Severino Italy; ^3^ U.O. Oncologia Azienda Ospedaliera Ospedali Riuniti Marche Nord Pesaro Italy; ^4^ U.O. Oncologia Ospedale A. Murri Fermo Italy; ^5^ U.O. Oncologia Ospedale E. Profili Fabriano Italy; ^6^ U.O. Oncologia Ospedale C. Urbani Jesi Italy; ^7^ U.O. Oncologia Presidio Ospedaliero Unico “Santa Maria della Misericordia” Urbino Italy; ^8^ U.O. Oncologia Ospedale di Civitanova Marche Civitanova Marche Italy; ^9^ U.O. Oncologia Ospedale di Macerata Macerata Italy; ^10^ U.O. Oncologia Ospedale di Senigallia Senigallia Italy

**Keywords:** cancer, cancer patients, coronavirus disease, COVID‐19, distress, oncology, psycho‐oncology

## Abstract

**Objective:**

The coronavirus disease 2019 (COVID‐19) outbreak has been declared a global pandemic of unprecedented proportions. Italy is a country which has been heavily affected. Cancer patients are at a higher risk owing to their intrinsic fragility related to their underlying disease and oncologic treatment. Against this backdrop, we conducted a survey to investigate how patients perceived their condition, clinical management and availability of information during the pandemic.

**Methods:**

Between 15 April and 1 May 2020 a survey was submitted to cancer patients at oncology departments in the Marche region. Questions regarding the perception of personal safety, continuity of cancer care, information quality and psychological distress.

**Results:**

Seven hundred patients participated in the survey; 59% were female and 40% were aged between 46 and 65. The majority of the participants perceived compliance with appropriate safety standards by cancer care providers and 80% were reassured about their concerns during the medical interview. 40% were worried of being at a higher risk of infection and 71% felt they were at a greater risk because of chemotherapy. 55% felt that postponing cancer treatment could reduce its efficacy, however 76% declared they did not feel abandoned at the time of treatment postponement. Patients between 46 and 65 years declared a significant reduction in sleep (*p* < 0.01) and in concentration (*p* = 0.03).

**Conclusions:**

The emergency care offered to cancer patients has been deemed satisfactory in terms of both safety standards and care management. However, the majority of participants perceived the mutual negative influence between their oncologic disease and the risk of infection highlighting the need for special measures to ensure safe continuity of care.

## 1 INTRODUCTION

During the coronavirus disease 2019 (COVID‐19) pandemic, cancer patients found themselves facing a double battle: fighting cancer and resisting the SARS‐CoV‐2 infection. Furthermore, they represent a high‐risk group due to their heightened susceptibility to infection, their underlying disease and their often immunosuppressed status. Therefore, severe complications from infections could develop.^1^ For patients infected by COVID‐19, treatment of the disease frequently takes priority over their cancer therapies, causing a delay of scheduled visits and treatments and the need to take decisions on a patient‐by patient basis.^1^, ^2^ Moreover, the inability to travel to hospital due to lockdown restrictions or difficulties in provision of medical care may result in a deterioration of mid‐ and long‐term outcomes of cancer treatment.^3^

The major cancer associations (American Society of Clinical Oncology, European Society of Medical Oncology, Associazione Italiana Oncologia Medica) have published recommendations to guide oncologists in the treatment of patients during the COVID‐19 pandemic, while ensuring their rights, safety and well‐being.^4^, ^5^, ^6^ Postponement or cancellation of non‐essential appointments, phone or web‐mediated consulting, electronic prescriptions to access home‐based cancer treatment (e.g., oral treatments) and delivery of medication are some of the common measures currently adopted.^7^

We therefore conducted a survey among cancer patients receiving treatment at Marche region oncology departments, aimed at exploring and establishing their perception of safety and of clinical and information management, their additional worries and any increase in distress during the COVID‐19 pandemic.

## 2 METHODS

Between 15 April 2020 and 4 May 2020, our team conducted a cross‐sectional study by submitting a survey to about 1100 patients with a diagnosis of solid cancer undergoing active treatments in oncology departments in the Marche region. Seven hundred patients participated in the survey; the others declined the investigation (about 10% declined the invitation to participate in the survey because of logistic/demographic or other unspecified reasons).

The oncology departments involved and the relative number of patients who participated in this survey are reported in Table 1. The survey consisted of 27 multiple choice questions, including 4 demographic questions, 12 questions regarding perception of personal safety and oncology department compliance with health and safety standards, 6 regarding perception of continuity and quality of care and 5 questions concerning personal psychological distress. All the questions were selected on the basis of patients' reported observations and complaints during medical visits in the initial phase of the pandemic.

**TABLE 1:** Demographic characteristics of survey respondents

Characteristics	Survey respondents (*N* = 700) no. (%)
Gender	
Female	413 (59.0%)
Male	276 (39.4%)
Not reported	11 (1.6%)
Age (years)	
≤25	2 (0.3%)
26–35	10 (1.4%)
36–45	45 (6.4%)
46–65	282 (40.3%)
66–75	229 (32.8%)
>75	131 (18.7%)
Not reported	1 (0.1%)
Educational level	
Degree	108 (15.4%)
High school diploma	261 (37.3%)
Lower secondary school diploma	206 (29.4%)
Primary school diploma	124 (17.7%)
Not reported	1 (0.2%)
Employment	
Pensioner	422 (60.3%)
Employee	144 (20.6%)
Freelancer	64 (9.1%)
Unemployed	67 (9.6%)
Not reported	3 (0.4%)
Oncology department	
Ancona	200 (28.6%)
San Severino	96 (13.7%)
Pesaro/Fano	87 (12.4%)
Fermo	84 (12.0%)
Senigallia	76 (10.9%)
Fabriano	43 (6.1%)
Jesi	38 (5.4%)
Urbino	28 (4.0%)
Civitanova	25 (3.6%)
Macerata	23 (3.3%)

The survey was submitted, in paper format, to all patients undergoing treatment at oncology departments at the time of access to Oncologic Units and collected at the time of discharge. The questionnaire was completely anonymous and did not collect sensitive data. The participation was voluntary after obtaining verbal informed consent.

According to Italian law (resolution 1 March 2012, Gazzetta Ufficiale n.72 of 26 March 2012), ethics approval was not required for the present study.

### 2.1 Statistical analysis

Demographic characteristics and answers were reported using relative frequency distribution.

The association between demographic characteristics and answers to questions regarding perception of personal safety, compliance with safety standards, continuity and quality of care and psychological distress was estimated by Chi square analysis. A level of 0.05 was chosen to assess the statistical significance. Statistical analysis was performed with STATA MP.11.0 software.

## 3 RESULTS

A total of 700 cancer patients participated in the survey. The majority of them were 46–65 years old (40%), more than 50% were over 65 (51%) and more than half of the patients involved were women (59%). Regarding educational level, 18% had only attended primary school, 29% and 37% had secondary and high school degrees respectively, and 15% had a bachelor's degree or higher (Table 1).

The majority of patients interviewed declared they had undergone the triage for suspected COVID‐19 symptoms (82%) when attending the Oncologic Day Hospital and they confirmed that the hospital complied with safety procedures (94%).

However, almost 40% of patients felt themselves to be at greater risk of contagion at the time of access to an oncology department, more than 70% perceived themselves and their family members at higher risk of infection compared to other people (71% and 73%, respectively), both due to the perceived need of systematic hospital access and immunosuppression induced by oncological treatment.

The majority of patients declared that oncologic professionals demonstrated competence in the COVID‐19 emergency and the instructions provided by healthcare staff were judged as homogenous (80% and 72%, respectively) (Table 2).

**TABLE 2:** Responses to questions concerning perception of personal safety and oncologic department compliance with safety standards

Question	Yes	No	Not reported
1. Have you been asked if you manifested symptoms which could be related to COVID‐19?	573 (81.8%)	100 (14.3%)	27 (3.9%)
2. Did your oncology department comply with the appropriate safety standards in this emergency?	661 (94.4%)	17 (2.4%)	22 (3.2%)
3. Have you been given any indications about personal protective equipment use upon entry in the oncology department?	611 (87.3%)	69 (9.8%)	20 (2.9%)
4. Did healthcare workers in the oncology department wear surgical masks and gloves?	669 (95.6%)	14 (2.0%)	17 (2.4%)
5. Did the staff demonstrate correct knowledge about COVID‐19 emergency?	505 (72.1%)	119 (17.0%)	76 (10.9%)
6. Do you think there is homogeneity of recommendations and indications provided by the healthcare staff in this emergency?	560 (80.0%)	68 (9.7%)	72 (10.3%)
7. Are you worried of being at higher risk of contagion?	495 (70.7%)	185 (26.4%)	20 (2.9%)
8. Are you worried that your family members could be at higher risk of contagion?	508 (72.6%)	172 (24.5%)	20 (2.9%)
9. Did you feel reassured about your concerns regarding COVID‐19 emergency during the medical interview?	524 (74.8%)	144 (20.6%)	32 (4.6%)
10. Do you feel more worried for your underlying condition in this emergency situation?	540 (77.2%)	138 (19.7%)	22 (3.1%)
11. Are you worried of being at higher risk of infection upon entry into the oncology department?	277 (39.6%)	393 (56.1)	30 (4.3%)
12. Have you been provided with information regarding the household management of new‐onset symptoms?	418 (59.7%)	234 (33.4%)	48 (6.9%)

Physicians and nurses were said to be easily contactable (by phone and/or email) according to the majority (88%) of patients interviewed, and 539 patients (77%) thought that during physical examination and medical interview an adequate amount of time had been dedicated to them in order to allow them to understand medical recommendations (Table 3).

**TABLE 3:** Responses to questions concerning perception of continuity and quality of care

Question	Yes	No	Not reported
	*No. of respondents (%)*		
1. Is the healthcare staff which has you in charge easily reachable by phone or email in this emergency situation?	618 (88.3%)	49 (7.0%)	33 (4.7%)
2. Have you been given enough time during the medical interview to understand the recommendations to be followed in this emergency?	539 (77.0%)	114 (16.3%)	47 (6.7%)
3. Does the healthcare staff give importance to your anxieties and concerns?	560 (80.0%)	84 (12.0%)	56 (8.0%)
4. Have you been provided with contact details of facilities to turn to in case the oncology department cannot cope with your needs due to emergency?	193 (27.6%)	428 (61.1%)	79 (11.3%)
5. Do you think that postponing a treatment in this emergency situation could compromise its efficacy?	388 (55.4%)	247 (35.3%)	65 (9.3%)
6. Did you feel a sense of abandonment when treatment or visit was postponed?	91 (13.0%)	529 (75.6%)	80 (11.4%)

They declared that medical staff gave importance to their concerns and fears and patients did not feel any sense of abandonment by healthcare professionals during the pandemic (80% and 76% respectively) (Table 3).

When assessing opportunities for improvement, more than half of interviewed patients (55%) felt worried about postponing scheduled treatments, believing this to be an issue that could compromise the efficacy of their treatment.

The majority of them (61%) complained that no alternative facility contact details were provided by the oncology departments in the event that the department would not be able to respond to their needs (Table 3).

Despite the perceived safe environment and the perceived homogeneity in medical recommendations, about one patient out three slept less, had a change in appetite and less ability to concentrate and reported a worse mood than usual (Table 4).

**TABLE 4:** Responses to questions concerning personal psychological distress

Question	Greater	Lower	Same	Not reported
	*No. of respondents (%)*			
1. Your sleep time is:	51 (7.3%)	238 (34.0%)	386 (55.1%)	25 (3.6%)
2. Your hunger is:	86 (12.3%)	142 (20.3%)	444 (63.4%)	28 (4.0%)
3. Your concentration ability is:	15 (2.2%)	208 (29.7%)	453 (64.7%)	24 (3.4%)
4. Your use of smoke and/or alcohol is:	15 (2.2%)	169 (24.1%)	328 (46.8%)	188 (26.9%)
5. Your mood is:	17 (2.4%)	232 (33.2%)	425 (60.7%)	26 (3.7%)

Statistical analysis did not reveal any significant association between concern about personal or family contagion and gender (*p* = 0.13 and *p* = 0.57, respectively), age (*p* = 0.49 and *p* = 0.47, respectively) or employment (*p* = 0.47 and *p* = 0.85, respectively). Similarly, there was no association between the perceived personal risk of contagion and the level of education (*p* = 0.34), however, those with lower levels of education were more frequently worried about risk of contagion to their family members than those holding a higher degree (*p* = 0.04).

Concerning personal psychological distress, no statistically significant correlations emerged between gender and change in sleep quantity (*p* = 0.07), appetite (*p* = 0.75) and ability to concentrate (*p* = 0.29), while significant differences were found between female participants and a higher consumption of nicotine and/or alcohol (*p* = 0.01) than usual and a worsening in mood (*p* = 0.01).

Patients who belonged to the 46–65 age group declared a significant reduction in sleep (*p* < 0.01), the ability to concentrate (*p* = 0.03) and an increase in nicotine and/or alcohol consumption (*p* < 0.01) compared to other age groups. There was no apparent significant impact on mood (*p* = 0.16).

When evaluating personal psychological distress responses in relation to the level of education, no significant correlation emerged in change in sleep patterns (*p* = 0.09), appetite (*p* = 0.46), the ability to concentrate (*p* = 0.35) and substance abuse (*p* = 0.26), but it emerged that those who have a degree experienced a significant worsening of mood than those who had a lower level of education (*p* = 0.04).

Finally, those in employment reported a significant change in sleep patterns and the ability to concentrate compared with the other categories (*p* < 0.01 and *p* = 0.02, respectively), with a trend of mood reduction (*p* = 0.05), but no significant change in substance abuse (*p* = 0.86).

## 4 DISCUSSION

By investigating the perspectives of cancer patients, our survey reveals that oncology departments were considered satisfactory in terms of providing care management safely during the COVID‐19 pandemic.

More than half of the patients interviewed were women. Several studies have shown that female participation in surveys tends to be higher than male participation, possibly owing to their different attitudes and interests.^8^

There were no other elements denoting imbalance in the sample if we consider the remaining demographic variables and general population features.^9^

Although 14% of cancer patients declared that they had not received a health triage, almost all (94%) patients felt confident of the clinical organization of the oncology departments in the Marche Region and they perceived staff as being adequately trained professionals capable of facing this unprecedented situation.

Furthermore, our survey demonstrated a conflicting scenario since 71% of patients felt at greater risk than the general population and stated that they were worried for their family members too, who could be at greater risk of contagion.^10^, ^11^

Cancer patients, particularly those who are in therapy, have been considered to be at increased risk of mortality from COVID‐19. This assumption had a great impact on scheduled treatment and on clinical reorganization and induced anxiety and fears in patients, as well as in oncology healthcare professionals.^7^, ^12^

At the time of attending the oncology department, 41% of patients expressed concerns of being at higher risk of infection upon entry and this emotional status could lead to a loss of compliance and to giving up attending the hospital to receive the necessary treatment which could lead to a potentially dangerous situation for the patient.^7^, ^13^

To the best of knowledge, there is no sufficient data about increased risk of mortality from COVID‐19 favored by anticancer treatments, hence scheduled administration should be respected in order to avoid a loss of benefit, in this difficult time.

Despite perceived safety and the absence of strong prognostic data, patients perceived a higher personal risk compared to other people^7^, ^13^, ^14^, ^15^ and this was reflected in a worsening of mood and a negative change in daily habits.

Overall, the most psychologically frail group was represented by female patients, aged between 46 and 65, and those in employment, confirming data from many other published sources in which supportive care program need emerged, in order to cope, especially for female patients.^16^, ^17^, ^18^, ^19^ There is support for the role of gender in other literature when considering the prevalence of psychological distress as women are consistently reported to be more distressed than men, partly due the division of responsibilities and privileges afforded to each sex in today's society. During this pandemic, these gender differences were highlighted in female patients and health workers.^20^

Results from patients with a lower level of education confirm that this is associated with worries and fears which are probably due to a lower health literacy and the consequent cognitive impact caused by inaccurate risk perception.^21^, ^22^

The cancer experience is portrayed as a continuous evolution of the disease and, with this, the emotional evolution of the subject who perceives himself as increasingly vulnerable in the cancer care continuum. Cancer patients reveal multidimensional needs over time, the components of which change during the course of treatment and as the disease progresses.^23^ The COVID‐19 pandemic has affected the National Health System through complex reorganization in the hospital setting, but in a similar way, the territorial healthcare system has been affected. It is evident that a significant gap in the continuity of care between hospital and territory emerged during the pandemic.^24^, ^25^

Hospital‐territory continuity of care should be mandatory considering that cancer care is required to be changeable and flexible, accommodating all of the imbalances which can be brought on by an emergency situation such as a pandemic.

Cancer diagnosis inevitably leads to the perception of a tunnel, of varying length, where the fear of dying, of suffering and the fear of treatments are real.^26^

The diagnosis leads to a change in perception of time and being and amplifies the feeling of mortality accompanied by a sense of cognitive and emotional solitude along the path.^24^

SARS‐CoV‐2 is an infection that causes isolation and in the case of a cancer patient testing positive would consequently lead to non‐treatment.^27^

Then, there are legitimate concerns around the postponement of treatment and the consequent loss of benefit.^25^ However, SARS‐CoV‐2 infection would also cause social distancing and isolation, in turn leading to a significantly heightened sense of loneliness and abandonment.^26^, ^28^

### 4.1 Study limitations

The major limitation of this study is the cross‐sectional design that implies the absence of a longitudinal perspective, which is undoubtedly of use when planning future strategic patient management. Furthermore, type of cancer and staging details are not available, this is another important limitation.

However, it is due to note that the survey was submitted during the peak of the Italian pandemic guaranteeing a reliable and accurate picture of the perception of cancer patients during the emergency.

### 4.2 Clinical implications

Even though our survey was conducted exclusively among cancer patients undergoing treatment in oncology departments in the Marche region, these results may be potentially generalizable considering that our region was one of the most affected by the COVID pandemic during the first wave in Italy.

Our investigation could help oncologists to improve the management of cancer patients during a second peak of SARS‐CoV‐2 infection as well as in future pandemics.

Considering the highly complex needs of cancer patients, including the psychological and emotional aspects, it is necessary to ensure continuity of care, making them feel safe through correct information and efficient clinical management.

## 5 CONCLUSIONS

As indicated from the findings, our survey revealed that we needed to do more to manage the array of needs presented by cancer patients during the COVID‐19 pandemic.

Although the oncology departments worked at high levels of safety, our survey underlined the importance of taking global care of cancer patients, from the management of their diseases, to the management of their fears, their perceptions of being a burden, as well as their feelings of aggravation and isolation. Inevitably, all these emotions are amplified by a health emergency like a pandemic and the oncologist has a fundamental role to fulfill in managing all of these elements, organic and emotional, because the cancer patient is not just an internist patient, but rather has an emotional and cognitive complexity caused by the nature of the disease.

## CONFLICT OF INTEREST STATEMENT

Rossana Berardi is a consultant/advisory board member for Astra Zeneca, Boehringer Ingelheim, Novartis, MSD, Otsuka, Eli‐Lilly, Roche.

All other authors declare that they have no conflicts of interest to declare.

## Data Availability

The data that support the findings of this study are available from the corresponding author upon reasonable request.
